# Motivation and social-cognitive abilities in older adults: Convergent evidence from self-report measures and cardiovascular reactivity

**DOI:** 10.1371/journal.pone.0218785

**Published:** 2019-07-10

**Authors:** Irene Ceccato, Serena Lecce, Elena Cavallini, Floris T. van Vugt, Ted Ruffman

**Affiliations:** 1 Department of Brain and Behavioral Sciences, University of Pavia, Pavia, Italy; 2 Department of Psychology, McGill University, Montréal, Quebec, Canada, Haskins Laboratories, New Haven, Connecticut, United States of America; 3 Department of Psychology, University of Otago, Dunedin, New Zealand; Educational Testing Service, UNITED STATES

## Abstract

Recently, some authors have suggested that age-related impairments in social-cognitive abilities—emotion recognition (ER) and theory of mind (ToM)—may be explained in terms of reduced motivation and effort mobilization in older adults. We examined performance on ER and ToM tasks, as well as corresponding control tasks, experimentally manipulating self-involvement. Sixty-one older adults and 57 young adults were randomly assigned to either a High or Low self-involvement condition. In the first condition, self-involvement was raised by telling participants were told that good task performance was associated with a number of positive, personally relevant social outcomes. Motivation was measured with both subjective (self-report questionnaire) and objective (systolic blood pressure reactivity—SBP-R) indices. Results showed that the self-involvement manipulation did not increase self-reported motivation, SBP-R, or task performance. Further correlation analyses focusing on individual differences in motivation did not reveal any association with performance, in either young or older adults. Notably, we found age-related decline in both ER and ToM, despite older adults having higher motivation than young adults. Overall, the present results were not consistent with previous claims that motivation affects older adults’ social-cognitive performance, opening the route to potential alternative explanations.

## Introduction

Empirical evidence has consistently shown that aging negatively affects social-cognitive skills, namely, emotion recognition and theory of mind. Emotion recognition (ER) refers to the ability to detect emotions as they are conveyed through different social stimuli, including facial expression, gaze direction, prosody of voices, bodily posture, and movements. Many studies have demonstrated an age-related decline in the ability to recognize emotions from facial, vocal, and bodily stimuli (for a review, see [1]). This decline has important implications for a variety of dimensions of social functioning, such as faux pas detection [2], verbosity [3], lie detection ability [4], and social attitudes [5]. Theory of mind (ToM) refers to the awareness that mental states (thoughts, beliefs, emotions) guide social behavior and allows people to predict, explain, and even affect others’ social behavior [6]. Therefore, ToM has significant consequences for everyday social interactions, social intelligence, and social competence [7–10]. A consistent body of research has found an age-related decline in ToM, irrespective of the type of task used (for a review, see [11]), and that appears partly related to general cognitive functioning [12,13].

Given that social-cognitive skills are necessary for successful social functioning [14], and social functioning is crucial for older people’s mental health [15], it is pivotal to understand which factors may modulate the effect of aging on social-cognitive abilities. In a shift from traditional research in this area that mainly considered the role of related cognitive abilities (e.g., executive functions) [13,16], in the present study we focused on motivation, i.e., the set of processes that influences people’s determination to carry out some task [17]. Understanding whether the drop-off in older peoples’ social-cognition reflects a true decline in this ability, or is (at least in part) due to a reduction in motivation, is important for several reasons. First, it may contribute to a more exhaustive model of variables underlying social understanding, thus allowing a better comprehension of older people’s social functioning. Also, from a theoretical point of view, if motivation can explain age-related decline in social-cognition, this result would need to be discussed in the light of findings indicating that diet (known to facilitate brain health) is related to older adults’ theory of mind [18], and that oxytocin (which facilitates neurotransmission) is related to older men’s emotion recognition [19]. Second, it would help change the understanding of older adults’ competence from a static view of social-cognitive skills to a more dynamic one, focusing not only on what people can do, but also on what they actually do. Further, if reduced motivation is responsible for weakened performances in older adults, intervention programs aiming at promoting social-cognitive skills should also target interest and effort, and not simply focus on the social-cognitive abilities per se.

In the current study we focused on motivation, by experimentally manipulating self-involvement, and thus effort mobilization in task accomplishment, and examined its effects on performances in ER and ToM tasks, in both young and older adults. Thus, in the following section we give a general overview of theoretical frameworks and empirical evidence concerning motivation and its role in cognitive performance of older people, and then those studies that specifically examined the relation between motivation and social-cognition.

### The role of motivation in older adults’ task performance

Motivation is a broad concept usually investigated in terms of goals, which are “cognitive representation[s] of a future object that the organism is committed to approach or avoid” [20]. That is, motivation refers to those processes that shape the direction and intensity of an individual’s behaviors [17]. Within cognitive-aging research, the role of motivation has been highlighted by the selective engagement theory [21,22]. According to this model, the cost to engage in cognitive activities increases with age. Therefore, older people reduce engagement in effortful activities, and carefully select where to put their resources, with personal goals preferred. For this reason, older people’s cognitive performance is argued to be disproportionately affected by the perceived significance of particular goals and motivational factors. Several studies have provided support for this model, showing that older adults’ performance on a wide range of tasks (e.g., memory and decision-making) is affected by experimental manipulations of motivation (for a review, see [22]). More generally, this line of research suggests that motivational factors play an important protective and compensatory function in aging, potentially explaining the paradox of successful everyday functioning, in spite of impaired cognitive performances in experimental tasks [22].

To date, the role of motivation in aging has been mostly investigated by examining memory, and there is a paucity of research in the social-cognitive domain; this seems a major gap considering the importance of social cognition for relational functioning and daily life. As far as we are aware, there are only three published studies that have directly investigated the impact of motivation—and more specifically, potential motivation, or justified effort—on ER and ToM in older adults. In the first study and the subsequent replication study, Zhang and colleagues manipulated familiarity (i.e., perceived closeness) between the subject and the experimenter. The authors took as their premise socioemotional selectivity theory [23], which argues that older adults’ reduced time-perspective leads them to emphasize emotionally meaningful goals over acquisition of information. Consequently, older people prioritize close partners over acquaintances as personally relevant, more than young adults. Hence, the authors hypothesized that the manipulation of perceived closeness may increase emotional meaningfulness and potential motivation in accomplishing a task, thus leading to better performance. In line with their expectations, the authors found that perceived closeness enhanced older Chinese participants’ performance on both ER and ToM tasks [24,25].

In the third study, Stanley and Isaacowitz [26] examined emotion recognition and empathic accuracy in an American sample. The authors operationalized motivation by manipulating the level of accountability (Study 1) or familiarity (Study 2). Accountability refers to the need to justify one’s judgments and decisions to others; it increases engagement in a task by making self-presentation concerns salient [27]. Familiarity was manipulated through task stimuli (i.e., judging the emotions of partners as opposed to strangers). The authors found a significant interaction between age group and accountability; however, follow-up tests within age groups indicated that older people in the high and low accountability conditions did not significantly differ, even if means were in the expected direction (82% correct versus 77% correct; p. 390). Concerning Study 2, the authors found that older adults were slightly better in the high familiarity condition (38% correct versus 29% correct), although still well below the level of young adults (61% correct).

In sum, previous findings provide some support for the idea that motivation explains older adults’ worse social understanding, yet there remain inconsistent findings. On the one hand, accountability should increase motivation and performance, yet Stanley and Isaacowitz [26] found that it did not significantly improve older adults’ emotion recognition performance. On the other hand, Zhang et al. [24,25] and Stanley and Isaacowitz [26] manipulated familiarity in markedly different ways, and found that it did improve older adults’ social-cognition.

More specifically, Zhang and colleagues operationalized familiarity in two ways. For ER ability, they compared close to not close people when administering a task in which participants had to judge the emotions of individuals (pictured in a red box). In the primed closeness condition, participants were told, “after checking your daily activity scale, I noticed that participants in the red box share a lot of common interests with you”, while in the primed distance condition, they were told that the target individuals, “share no common interest with you; you have very distinct interests from them”. These instructions essentially create either an “in-group” (a social group to which a person identifies psychologically as being a member) or it’s opposite, an “out-group”. Research shows that people from diverse cultures (Japan, America, Chile) are better at judging the emotions and mental states of individuals of their in-group compared to an out-group [28–30]. Thus, by identifying individuals “in the red box” as either in-group or out-group members, Zhang et al. would either facilitate or depress emotion recognition because out-groups are subject to greater dehumanization [31]. Moreover, it might be that older adults are more susceptible to in-group/out-group biases than younger adults, consistent with the idea that older adults are more susceptible to prejudice [32], and that dehumanization of the out-group rather than lowered motivation helps to explain worse emotion recognition.

The second way that Zhang et al. [24,25] primed closeness was twofold: first, the experimenter told participants that the participant’s answers on a daily activity scale were very similar to the experimenter’s own profile; second, a participant’s relative (grandchild or sibling) served as experimenter. Thus, in both cases, the primed closeness manipulation created an in-group containing experimenter and participant. Participants were then given two tasks tapping social-cognitive understanding, judging whether protagonists in written social scenarios had made a faux pas, and interpreting the mental states of geometric shapes moving on a screen, with performance better in the primed closeness condition compared to a standard condition. Yet, once again, something other than motivation may have affected the results. Research indicates that perceived similarity lowers stress [33]. This is especially important for an older adult who, relative to a young undergraduate, has little experience of coming into a university lab and being tested through tasks designed to examine social-cognition. Thus, the testing scenario is liable to be more stressful for older adults, but perceived closeness might lower stress and improve performance.

Finally, Stanley and Isaacowitz found that older adults were better when labeling the emotions of familiar others (partners) compared to strangers, and they attributed their findings to enhanced motivation when judging the emotions of familiar others (Study 2, [26]). Nevertheless, Stanley and Isaacowitz did not directly measure motivation to establish that it was the mediating factor, which raises the possibility that familiarity might have affected performance for a different reason. Indeed, based on their years together as a couple, older adults had more than 17 times as much experience with their partners compared to young adult experience with their partners. As such, older adults had many more years to learn to identify subtleties in their partners’ emotional expressions. Thus, rather than perceived closeness increasing emotional meaningfulness and potential motivation, greater experience and learning about partners’ expressions may have facilitated older adults’ performance when judging the emotions of familiar others.

In sum, both Zhang and colleagues [24,25] and Stanley and Isaacowitz [26] argued that familiarity facilitates older adults’ motivation and subsequent social-cognitive reasoning. However, neither group actually measured motivation and it is possible to hypothesize different explanations for each finding not relying on motivation and effort mobilization.

When motivation is assessed, the most frequent way to measure it is through self-report questionnaires. However, recent findings have shown the benefit of using physiological indices to measure potential motivation [34]. Research shows that a high level of motivation leads to greater engagement and effort [35], and therefore, can be detected with blood pressure measures, such as systolic blood pressure (SBP). Systolic blood pressure refers to the pressure against artery walls following a heart beat, reflecting sympathetic nervous system activation. Specifically, the *change* in SBP (SBP-Reactivity or SBP-R) during task accomplishment reflects cognitive resource mobilization, related to task difficulty, ability, and motivation [36–38]. Notably, in the field of aging, the few studies investigating motivation with cardiovascular reactivity have indicated that when motivation is tested with the objective physiological measure, SBP-R, older adults show *higher* effort and engagement levels than young adults across a range of different tasks and difficulty levels [39,40].

### Present study

Previous research is intriguing in suggesting that motivation might affect ER and ToM, although findings are open to alternative interpretations other than motivation. The present study set out to overcome two major limitations. First, in previous studies there was no measure of motivation, either a self-report questionnaire or an objective physiological measure such as blood pressure. In the present study, we used both types of index, taking measures of motivation at baseline, and after the motivation manipulation.

Second, previous studies did not examine whether a positive effect of motivation would be specific to social-cognitive performance (i.e., ER and/or ToM), or whether it extended to general task performance. Zhang and colleagues [24,25] and Stanley and Isaacowitz [26] have argued that motivation affects performance on social-cognitive tasks. However, according to the selective engagement theory older adults should refrain from engaging in effortful activities [22], and this should affect their performance on a range of tasks, including non-social control tasks. Thus, in the present study we used both social-cognitive tasks and control (non-social) tasks that were similarly structured to the social-cognitive tasks. Specifically, we gave participants a task, the Matching task, which measured emotion recognition ability (matching emotion sounds and faces), but also included control items (matching non-emotion sounds and objects). We also gave participants a ToM task, in which participants described the apparently intentional and inter-connected movement of two triangles (e.g., one “fooling” the other), as well as control items (describing the random movement of two triangles). In this way, we could test whether an effect of effort mobilization is generalized across tasks, thus indicating a generic positive effect on task involvement and performance, or rather, that motivation specifically impacts on social-cognitive performance.

We studied the effect of potential motivation by manipulating the level of self-involvement when taking part in social-cognitive tasks. Self-involvement (also called *ego-involvement*) refers to the importance given to a task in terms of the personal commitment to obtain the best possible performance [41]. According to the motivational intensity theory [42], when the difficulty of a task is unclear, or when the task is considered hard but not impossible, self-involvement enhances effort mobilization and performance [41,43]. Previous research shows that a self-involvement manipulation successfully affects memory, concentration, and emotional bias susceptibility in young adults [41,44,45], but it has never, to the best of our knowledge, been investigated in older adults. However, there are reasons to think that a self-involvement manipulation would be effective for older adults. Indeed, the definition of self-involvement seems to overlap, at least in part, with notions about both self-relevance and socio-emotional goals, widely investigated in the aging literature [46,47]. For instance, Klein and Schoenfeld argue that a high level of self-involvement means that “important ego-factors (e.g., social prestige, self-esteem, fear of academic standing) are closely bound up in the tasks, and […], because of this, performance is of more vital consequence to the subjects” (p. 249, [48]).

Hence, in the current study we randomly assigned young and older participants to either a High self-involvement condition in which they were told that their performance on the tasks tapping social-cognition was associated with several desirable social outcomes such as positive relationships, better communicative skills and wisdom. Alternatively, in a Low self-involvement (control) condition, they were given general information about how and when the tasks were created. Along with a subjective evaluation of motivation (self-report questionnaire), we measured blood pressure repeatedly throughout testing as an objective index of effort mobilization, expecting that it would increase to a greater extent in the High self-involvement condition than in the control condition, reflecting higher motivation.

If motivation is important for improving older adults’ social-cognitive performance as previous studies suggested, we expected that the increase in motivation following the self-involvement manipulation would lead to a greater improvement in older adults’ ER and ToM performance than for young adults. With respect to the control (non-social) items, we hypothesized a positive effect of greater self-involvement in both young and older participants, as motivation has been shown to relate to cognitive performance on tasks tapping memory, planning, and decision-making in both age groups (for a review, see [22,49]). Therefore, we would expect older adults’ self-reported motivation and SBP-R to be higher in the High self-involvement condition than the Low self-involvement condition, and if so, their performance should improve in the High self-involvement condition.

In contrast, if potential motivation is not such a crucial determinant of older people’s social-cognitive performance, we would expect to find little effect of the self-involvement manipulation on socio-cognitive performance. Furthermore, when examining individual differences in the older adult group, we would expect to find absent or weak associations between motivation and social-cognitive performance.

Finally, because of the highly verbal nature of the ToM task, we gave participants measures of their general language ability to determine whether this mediated performance.

## Materials and methods

### Participants

The initial sample comprised 64 older and 66 young adults randomly assigned to either the experimental condition, in which motivation was manipulated (High self-involvement condition), or to a control condition (Low self-involvement condition). Three participants (two old, one young) were excluded from the analyses because the initial screening of SBP revealed values exceeding 160 mmHg [40]. In addition, one young participant was excluded because of anomalous CV-R values (namely, a rise in SBP during the Matching task of more than 3 *SD* relative to the other younger participants, as well as a decrease in diastolic blood pressure and an increase in heart rate during both the Matching and the Animation tasks of more than 2 *SD* compared to the other young participants). One additional older participant was excluded because her self-reported motivation was more than 3 *SD* below the average. Five young adults were excluded because English was not their native language and two more were excluded due to procedural errors during the administration.

Thus, the final sample comprised 61 older adults (age: *M* = 73.90, *SD* = 4.53, range 65–85; male = 34%; 29 in the Low self-involvement condition and 32 in the High self-involvement condition), and 57 young adults (age: *M* = 20.61, *SD* = 3.22, range 18–35, male = 25%; 28 in the Low self-involvement condition and 29 in the High self-involvement condition). Excluded participants did not differ from the final sample in age, education, or verbal reasoning, all *Fs*(1,64) ≤ 1.82, *p* ≥ .182. The one exception was that excluded young adults had lower verbal knowledge than young adults in the final sample, because they were not English native speakers, Welch’s *F*(1,9) = 8.59, *p* = .017.

We used a fixed-sample stopping rule, collecting data to reach about 30 subjects in each condition for both young and older adults. An after-the-fact power analysis [50] focusing on the main research aim, namely the interaction effect between Age Group (young vs. old) and Condition (high vs. low self-involvement) on performance, was carried out using G*Power 3.1.9.2 [51]. Based on previous studies which reported a medium effect size [26], and *α* = .05, we found that with 118 participants we had enough power (1-*β* = .84) to detect a medium effect.

Older adults were volunteers recruited from the community. All participants scored above the 82-point cut-off for dementia screening on the ACE-R (Addenbrooke’s Cognitive Examination—Revised; [52]), *M* = 94.93, *SD* = 3.97, were stroke-free, and reported no neurological insults. Information about medications that may affect blood pressure was collected, with 52.5% of participants reporting that they were under treatment for hypertension (see below for analyses comparing those on, and not on, blood pressure medication). Participants were reimbursed $10 to cover their travel expenses. Young adults were psychology students who received partial course credit for writing a report detailing their participation. They reported no neurological insults, nor were they taking medication affecting blood pressure.

### Measures

#### Emotion recognition

To measure emotion recognition ability we used the Matching task [53,54]. In this task, six pictures were presented on a computer screen, each one identified with a number. Participants listened to a soundtrack and selected the corresponding picture. The task includes two parts: emotion recognition and control (non-emotion). The emotion recognition task contained 12 emotional soundtracks that participants heard twice (though never consecutively). Soundtracks comprised both sentences pronounced with different prosody—six soundtracks—and expressive sounds (like a happy humming sound or high-pitched gasps of fear—six soundtracks). The participant’s task was to match the soundtrack to one of six facial expressions portraying the six basic emotions (happiness, sadness, anger, surprise, disgust, and fear). All emotions were portrayed by “JJ”, one of 10 individuals in the Ekman and Friesen emotion recognition battery. Each emotion sound was presented four times, twice as a sentence and twice as an expressive sound, for a total of 24 items. Reliability on this task was acceptable, Cronbach’s *α* = .71.

The control (non-emotion) task contained 24 soundtracks, comprising four categories of sounds (i.e., birds, vehicles, gardening, and household appliances). Six non-emotion photos (e.g., household appliances) were shown on the computer monitor and the participant’s task was to match each soundtrack to the appropriate picture, when presented with six different pictures of the same category (e.g., the sound of a sewing machine to the image of a sewing machine, with distractors including a fridge, a washing machine, an electric shaver, a vacuum cleaner and a blender).

The Matching task was chosen instead of a standard facial expression test because: (a) it tends to be a more difficult and sensitive measure of emotion recognition [1], (b) it provides a more comprehensive measure of emotion recognition in that it measures both facial and auditory emotions rather than just facial emotions, and (c) it can be compared to a corresponding control task. The two parts of the task were included in a single battery, with emotion and non-emotion items randomized and the order of items held constant across participants. Total scores were created by separately summing emotion and non-emotion items, thus obtaining two different scores (emotion and non-emotion control score), both ranging from 0 to 24.

#### Theory of mind

The Animation task [55,56] required participants to describe short videos depicting two triangles moving about on a screen. The task was composed of three types of animations: (1) Random, in which triangles moved randomly (e.g., bouncing, rolling), (2) Goal-directed, in which triangles moved in a goal‐directed fashion (e.g., chasing, fighting), and (3) Cognitive, in which triangles moved again with goals, but also with more complex interactions implying the manipulation of mental states (e.g., pretending or tricking). The Cognitive videos were used as the ToM measure because they required the highest level of mentalistic reasoning (cognitions rather than just goals).

We used eight animations for each type for a total of 24 videos. The order of presentation of the videos was randomized and held constant across participants. Before the start of the task, two examples were given (a Random item and a Cognitive item) followed by an example of a description that focused on the differences between the two videos, pointing out the belief manipulation in the second one. Each animation lasted approximately 15 seconds and at the end of each one participants were asked, “What was happening in this animation?” Answers were audio-recorded and subsequently coded according to the degree to which participants described complex, intentional mental states. Intentionality scores for each item ranged from 0 (no purposeful action) to 5 (one shape trying to manipulate the mental states of another shape). Total scores for each type of video ranged from 0 to 40. Coders were blind to participants age and condition. Inter-rater reliability was assessed by computing a two-way random, absolute agreement, average-measures intraclass correlation coefficient (ICC). Inter-rater reliability was .79, a level deemed excellent [57].

#### Language

Verbal abilities were measured with two tests: the Similarities subtest of the WAIS-IV [58] and the Shipley vocabulary test [59]. The Similarities test required participants to describe how two presented words (common objects or concepts) were similar; it is a measure of verbal concept formation and reasoning, measuring both receptive and expressive language. Scores ranged from 0 to 36. The vocabulary test consisted of 40 items in which participants provided the correct synonym of a given word from amongst four choices within 10 minutes. It is a measure of general linguistic knowledge and receptive language ability. Scores ranged from 0 to 40.

#### Motivation

**Self-report questionnaire.** This questionnaire included five items investigating motivation, interest and effort in performing the task. Answers were given on a Likert scale ranging from 1 (*not at all*) to 7 (*very much*). The questionnaire was created by adapting scales previously used in the literature [44,60,61]. A global self-reported motivation score was computed by averaging the five items separately for the two tasks. Internal consistency was, α = .70 and α = .74, respectively for the Matching and Animation tasks.

**Cardiovascular indices.** We measured cardiovascular values with an automatic blood pressure monitor (Sanitas, model SBM 21) that uses oscillometry to determine systolic blood pressure (SBP—millimetres of mercury [mmHg]), diastolic blood pressure (DBP—mmHg), and heart rate (HR—beats per minute). Hereafter, we report exclusively SBP results because previous studies suggested that SBP is an accurate index of effort and motivation [34], although we note that the main results were the same regardless of whether using SPB, DBP or HR. Analyses on DBP and HR are reported in the supplementary material (S1 File). A blood pressure cuff was placed over the brachial artery above the elbow of a participant’s non-dominant arm throughout the experiment. The cuff inflated automatically when the experimenter pressed a button. Cardiovascular measures were taken at regular intervals of 5 minutes during the rest phase (i.e., baseline values) and the two tasks phases (i.e., Matching task and Animation task).

Baseline values were collected during a 10-minute habituation period at minutes 0, 5 and 10 (with the first measurement used only to acclimatise participants to the device, but not contributing to the baseline value). One additional measurement was taken while participants filled in the Social network questionnaire. Cardiovascular values obtained through these three readings were then averaged into a baseline systolic blood pressure value (SBP-base).

Similarly, three cardiovascular readings were taken during each of the two task phases, starting within 30-60s after the task’s onset [62]. For 23 participants (11 young, 12 older) during the Animation task, only two CV readings were collected instead of three, due to rapid task completion. Thus, for these participants, only two values contributed to the average score on this task). Again, these readings were averaged to obtain a single measure of blood pressure during task accomplishment, namely SBP-Matching and SBP-Animation.

Cronbach’s alphas were .82 for the baseline and .91 over the Matching and Animation task performance. To control for individual differences in physiological indices, we computed cardiovascular change (delta) scores as a measure of cardiovascular reactivity [60,63], by subtracting the baseline value from the values obtained during either the Matching or Animation task. Nevertheless, we note that using residualized change scores instead of delta scores led to the same pattern of results.

### Procedure

The data were collected after obtaining the approval of the University of Otago Human Ethics Committee. Participants were tested individually in a quiet room in the Psychology Department. Written informed consent (asking also for age and educational level) was obtained before the testing session, which lasted approximately 1-^1^/_2_ hours. First, participants were asked to relax during a 10-minute habituation period, during which baseline SBP was taken. Some magazines were available to pass the time. Participants then completed the tasks in the following fixed order: Social network questionnaire (not relevant to the present study and therefore not discussed further), Matching task, Similarities test, Animation task, and Shipley vocabulary test.

The High self-involvement and the Low self-involvement conditions differed exclusively on the instructions given prior to administration of the Matching and Animation tasks. Participants in the High self-involvement condition read instructions stressing that performing well in the task was related to social competence and interpersonal wellbeing and that at the end of the experiment they would receive their results. Thus, this condition was designed to raise the level of self-involvement because the task was presented as a means to demonstrate the possession of important social skills relevant to people’s socio-emotional life. Also, offering personal results will further raise motivation, by increasing people’s perceived utility of the assessment. Participants in the Low self-involvement condition read instructions focused on the task itself, and thus, received information concerning how the task was created. Instructions in both conditions were matched in length (see the S1 Appendix). Note that we manipulated motivation twice, i.e., before each task. This was done as previous findings suggested that people may forget the manipulation contained in the instruction, and we want to avoid that any potential effect of our manipulation could degrade over time. The self-report motivation questionnaire was presented twice, once after the Matching task and once after the Animation task. Finally, participants completed the Shipley vocabulary test and were debriefed and thanked. Participants in the High self-involvement condition were also informed that they would receive their results through email/mail as soon as data collection ended.

### Statistical analyses

Raw data are available in S1 Dataset. We first examined differences between young and older participants across conditions in background variables (i.e., age, education, language ability) and SBP at baseline. We then investigated the effect of medical treatments on SBP reactivity in older adults. Next, we tested the efficacy of the manipulation of motivation examining differences between conditions in self-reported motivation, and SBP-R. To test the effectiveness of the motivation manipulation, we also computed Bayes factors (*B*_10_) that allowed us to test the evidence in favor of either the alternative or the null hypothesis. In the present study, the alternative hypothesis (H_1_) corresponds to motivation (either self-reported or measured with SBP-R) being greater in the High self-involvement than the Low self-involvement condition, whereas the null hypothesis (H_0_) corresponds to equal motivation in the High self-involvement and Low self-involvement conditions. Given this directional prediction of the effect, H_1_ was modeled as a half-normal distribution. Thus, in the Bayes factor, *B*_H(0,x)_ the H indicates a half-normal, the 0 indicates its mode is 0 and the x indicates the standard deviation, where x represents the roughly expected effect size based on relevant past studies. Previous studies using the self-involvement manipulation showed a mean difference between the High and Low self-involvement conditions in self-reported motivation of about 13% [41,45,60], which would equate to 0.93 points on a 7-point Likert scale, as we used in the present study. Concerning SBP-R, past studies found a mean increase of 7 mmHg in the High self-involvement condition relative to a Low self-involvement condition, and thus we used this value as the expected effect size [41,43,60]. Following Dienes [64], we considered Bayes (*B*) values above 3 indicative of substantial evidence for the H_1_ over H_0_, whereas *B* values below 0.33 indicated substantial evidence for H_0_ (the null hypothesis) over H_1_. *B* values between 0.33 and 3 reflect data insensitivity in distinguishing the null and alternative hypotheses. Bayes factors were computed with the Dienes’ online calculator (http://www.lifesci.sussex.ac.uk/home/Zoltan_Dienes/inference/Bayes.htm).

We also compared social-cognitive performance (emotion recognition on the Matching task and cognitive videos on the Animation task), as well as performance in the corresponding control (non-social) tasks, in the High self-involvement versus the Low self-involvement conditions.

Finally, we computed correlations separately for young and older adults to examine whether individual differences in motivation (both in terms of the self-report measure and SBP-R) were associated with differences in social-cognitive performance. We also computed *B* factors for particular correlation analyses. The alternate hypothesis (H_1_) corresponds to motivation (either self-reported or measured with SBP-R) being positively and moderately related to performance [41,65]. Therefore, we modeled H_1_ as a uniform distribution ranging from 0 to 0.549, indicating expected Pearson’s *r* values from 0 to 0.50.

## Results

### Preliminary analyses

Descriptive statistics for age, gender, education, language ability, and SBP-base, are presented in Table 1. We used separate 2 (Age Group: young, older) x 2 (Condition: high self-involvement, low self-involvement) analyses of variance (ANOVAs) for each one of the variables listed in Table 1 (except gender, for which we used chi-square analyses). In line with the aging literature, there was a significant main effect of age group on both education, *F*(1, 112) = 16.16, *p* < .001, $\eta_{p}^{2}=.13$, and verbal knowledge, *F*(1, 113) = 159.29, *p* < .001, $\eta_{p}^{2}=.58$, with older adults having higher scores than young adults. Also, older adults had a higher SBP-baseline measure, *F*(1, 114) = 52.60, *p* < .001, $\eta_{p}^{2}=.32$. Note that all *F*s were significant after controlling for multiple analyses using Holm’s correction to ensure the family-wise error was *p* < .05. No age differences emerged for verbal reasoning, *F*(1, 114) = .29, *p* = .593. Additionally, neither a main effect of condition, nor an Age Group x Condition interaction, was significant for any of the dependent variables (all *F*s ≤ 2.04, all *p*s ≥ .156). Furthermore, there were no differences in gender distribution across the samples, as a function of age group, χ^2^(1) = 1.37, *p* = .314, or condition, χ^2^(1) < 0.01, *p* = 1.00. Finally, there was no difference in participants’ ages for those assigned to the High or Low self-involvement conditions: young adults, *F*(1, 54) = 0.28, *p* = .600; older adults, *F*(1, 59) = 1.18, *p* = .282.

**Table 1:** Means (and *SD*) of background variables and SBP at baseline, as a function of age group and condition.

	Young			Older		
	*Low* *self-involvement*	*High* *self-involvement*	*Tot*	*Low* *self-involvement*	*High* *self-involvement*	*Tot*
**Age**	20.37 (2.75)	20.83 (3.64)	20.61 (3.22)	73.24 (3.84)	74.50 (5.06)	73.90 (4.53)
**Gender**	1.75 (0.44)	1.76 (0.44)	1.75 (0.43)	1.66 (0.48)	1.66 (0.48)	1.66 (0.48)
**Education**	3.15 (0.36)	3.14 (0.45)	3.15 (0.41)	3.79 (1.05)	3.81 (1.23)	3.80 (1.14)
**Verbal reasoning**	27.25 (4.62)	26.28 (4.16)	26.75 (4.38)	26.14 (5.13)	26.50 (4.06)	26.33 (4.56)
**Verbal knowledge**	28.82 (3.71)	30.00 (3.49)	29.42 (3.62)	36.66 (2.22)	36.74 (2.89)	36.70 (2.57)
**SBP-base**	119.38 (10.17)	112.80 (11.83)	116.04 (11.44)	133.76 (14.47)	133.44 (14.99)	133.59 (14.63)

*Note*. SBP-base = systolic blood pressure during the baseline phase. Educational level was categorized on a six-point scale (1 = up to fifth grade; 2 = high school, no diploma; 3 = high school diploma; 4 = bachelor’s degree; 5 = master’s degree; 6 = doctorate degree or professional degree).

Next, we examined the role of blood pressure medications for older participants. Based on participants’ reported medications, we considered four levels: no medication (47.5% of participants), diuretic or statin (8.2%), ACE-r or calcium-channel blocker (21.3%), and beta-blocker (23%). The High and Low self-involvement conditions did not differ in participants’ frequency of medication consumption, *χ*^*2*^(3) = 2.30, *p* = .550. Additionally, we tested whether medication consumption impacted on SBP reactivity when collapsed across the Matching and Animation tasks. Medication (four levels as described above) was the independent variable. Although medication likely depressed SBP overall, it had no effect on the reactivity measure, *F*(3, 57) = 0.50, *p* = .682. For this reason, we did not exclude participants based on their medication consumption.

### Manipulation of motivation check

We then tested whether the self-involvement manipulation increased motivation for older adults. First, we examined self-reported motivation across the Matching and Animation tasks (with means and standard deviations presented in Table 2) using a mixed 2 (Age Group: young, older) x 2 (Condition: high self-involvement, low self-involvement) x 2 (Task: matching, animation) ANOVA. Age Group and Condition were between-subjects variables and Task was a within-subjects variable. The dependent variable was participants’ self-reported motivation. There was a significant main effect of Age Group, indicating that older adults were more motivated than young adults, *F*(1, 114) = 17.83, *p* < .001, $\eta_{p}^{2}=.14$. However, neither the main effect of Condition, *F*(1, 114) = 0.73, *p* = .394, nor the Condition x Age Group interaction, *F*(1, 114) = 0.24, *p* = .627, were significant. Likewise, the three-way Condition x Age Group x Task interaction was not significant, *F*(1,114) = 0.54, *p* = .462. To better understand the lack of an effect for Condition, we used a Bayesian analysis to determine whether we could accept the null hypothesis of no condition difference. We found that *B*_*H*(0,0.93)_ = 0.038, indicating support for H_0_, namely that the two conditions did not differ in self-reported motivation.

**Table 2:** Means (and *SD*) of self-reported motivation, SBP-R, and social-cognitive performance, as a function of age group and condition.

	Young			Older		
	*Low* *self-involvement*	*High* *self-involvement*	*Tot*	*Low* *self-involvement*	*High* *self-involvement*	*Tot*
**Matching task**						
Self-reported motivation	4.78 (1.05)	4.99 (0.96)	4.88 (1.00)	5.39 (0.82)	5.63 (0.59)	5.51 (0.72)
SBP-R	-0.93 (7.80)	-1.47 (7.12)	-1.20 (7.40)	2.88 (8.97)	2.59 (9.86)	2.73 (9.37)
Emotion recognition	16.50 (2.74)	16.59 (3.20)	16.54 (2.96)	13.36 (3.65)	13.61 (3.42)	13.49 (3.51)
Non-emotion control	16.96 (2.52)	17.59 (2.41)	17.28 (2.34)	17.46 (3.25)	16.32 (2.99)	16.86 (3.14)
**Animation task**						
Self-reported motivation	4.68 (1.11)	4.59 (1.17)	4.63 (1.13)	5.24 (0.83)	5.42 (1.03)	5.33 (0.94)
SBP-R	5.33 (11.85)	1.68 (7.32)	3.48 (9.89)	9.38 (11.58)	5.76 (12.12)	7.48 (11.91)
Cognitive videos	28.85 (5.52)	31.21 (3.73)	30.07 (4.79)	25.93 (4.81)	24.77 (5.01)	25.33 (4.91)
Goal-directed videos	19.96 (3.06)	22.07 (3.68)	21.04 (3.53)	23.52 (5.45)	22.74 (4.22)	23.10 (4.80)
Random videos	1.00 (1.41)	3.48 (3.84)	2.26 (3.15)	10.69 (7.23)	12.75 (5.76)	11.83 (6.48)

SBP-R = systolic blood pressure reactivity (i.e., task value—baseline value).

We then used an identically structured 2 (Age Group) x 2 (Condition) x 2 (Task) ANOVA to analyze SBP-R as an index of motivation (see means and *SD*s in Table 2). No effects for Condition (main effect or interactions) were significant (all *F*s ≤ 2.81, all *p*s ≥ .097). Yet, once again, there was a main effect for Age Group (with older adults having higher SBP-R), indicating that older adults mobilize more effort than young adults, *F*(1, 114) = 6.82, *p* = .010, $\eta_{p}^{2}=.06$. Similar to the findings for self-reported motivation, the main effect of Condition revealed that *B*_*H*(0, 7)_ = 0.009, indicating strong evidence in favor of the null hypothesis, that is, no difference in effort mobilization between conditions.

To summarize, we found evidence in favor of H_0_, namely that the experimental manipulation of self-involvement did not increase self-reported motivation, nor did it impact SBP-R, which is associated with higher effort mobilization. However, there were still interesting effects for motivation in that the self-report measure and physiological SBP-R measure were consistent in indicating *higher* motivation in older adults relative to young adults.

### Social-cognitive performance

The main aim of the present study was to investigate the effect of motivation on social-cognitive performance. Table 2 presents the means and standard deviations for the social-cognitive measures. We first analyzed the Matching task using a 2 (Condition: high self-involvement, low self-involvement) x 2 (Age Group: young, older) x 2 (Task Type: emotion recognition, non-emotion control) mixed ANOVA, with Condition and Age Group as between-subjects variables and Task Type as a within-subjects variable. The dependent variable was the score out of 24 on the emotion and non-emotion control tasks. Main effects of Age Group and Task Type emerged, and were qualified by a significant Age Group x Task Type interaction, *F*(1, 112) = 14.98, *p* < .001, $\eta_{p}^{2}=.12$. Replicating Sullivan and Ruffman [66], univariate analyses showed that older adults performed significantly worse on the emotion recognition task, *F*(1, 112) = 25.23, *p* < .001, $\eta_{p}^{2}=.18$, but not on the non-emotion control task, *F*(1, 112) = 0.55, *p* = .458, compared to young adults. No other effects reached statistical significance (all *F*s ≤ 1.95, all *p*s ≥ .165).

**Fig 1:**
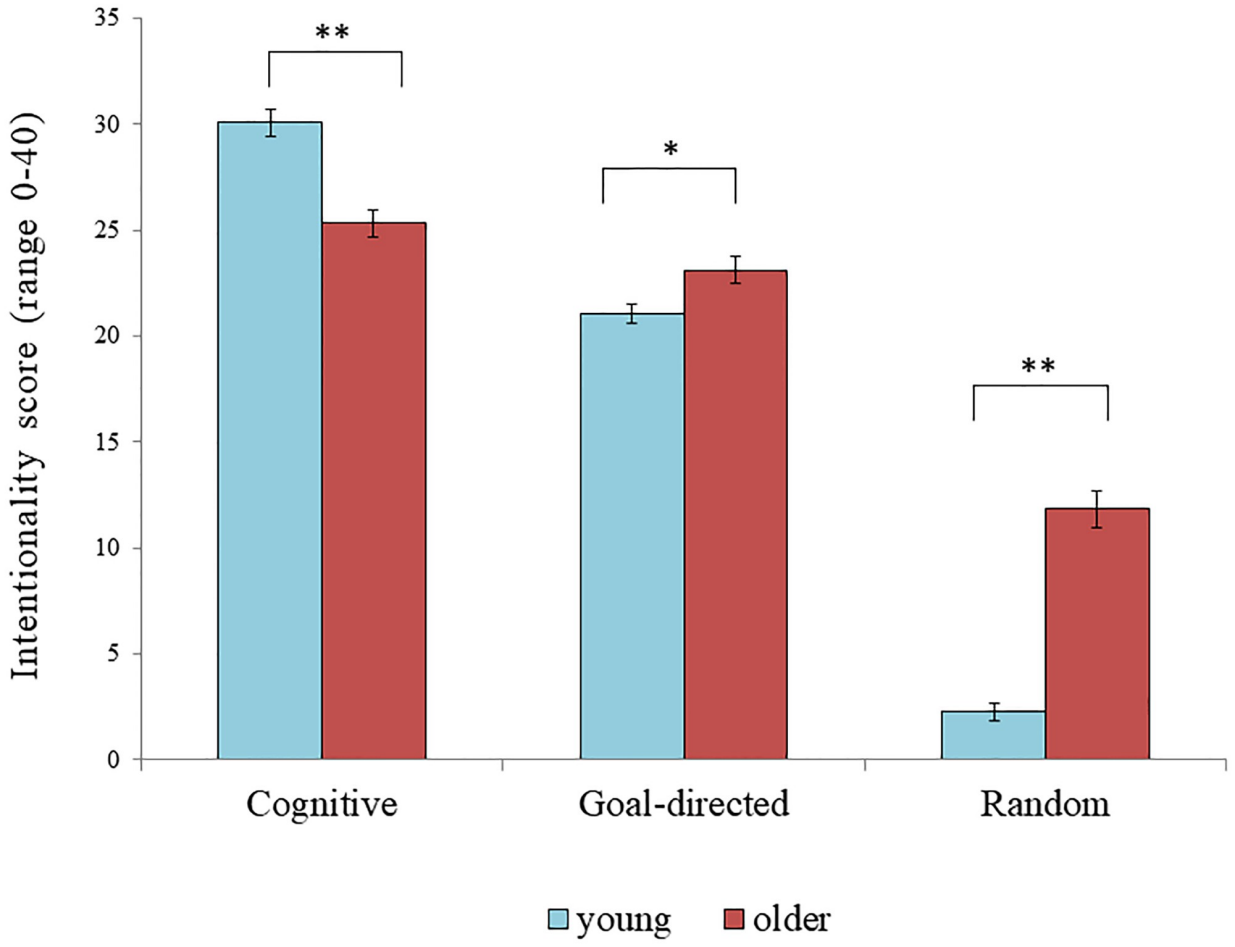
Intentionality score in the animation task as a function of age group and type of video. Error bars represent standard errors. Reported results refer to *t*-tests with Holm-Bonferroni’s adjustment. *Note*. * *p* < .05, ** *p* < .001.

We then analyzed the Animation task, comparing the three types of videos using a 2 (Condition) x 2 (Age Group) x 3 (Video Type: Cognitive, Goal-Directed, Random) mixed ANOVA, with Condition and Age Group as between-subjects variables and Video Type as a within-subjects variable. The dependent variable was the Intentionality score out of 40. As expected, there was a main effect of Video Type, *F*(2, 107) = 711.75, *p* < .001, $\eta_{p}^{2}=.93$, indicating that the amount of attributed intentionality differed depending on the type of animations (Cognitive > Goal-directed > Random). Neither the main effect of Condition, nor the Condition x Video Type, or the Age Group x Condition x Video Type interactions were significant, all *F*s < 2.67, all *p*s > .105. The Age Group x Condition interaction was marginally significant, *F*(1, 108) = 2.91, *p* = .091, $\eta_{p}^{2}=.03$. Interestingly, there was a main effect of Age Group, indicating that older adults had a significantly higher Intentionality score than young adults, *F*(1, 108) = 11.85, *p* = .001, $\eta_{p}^{2}=.10$, that was qualified by a significant Age Group x Video Type interaction, *F*(2, 107) = 77.22, *p* < .001, $\eta_{p}^{2}=.59$. The interaction was explored with *t*-tests (with Holm-Bonferroni adjustment; [67]), comparing young and older adults’ intentionality scores for each video type. Young adults had a higher Intentionality score than older adults on Cognitive animations, *t*(114) = 5.26, *p* < .001, while older adults had a higher Intentionality score than young adults on both the Goal-directed and Random animations, respectively, *t*(113) = 2.63, *p* = .010 and *t*(113) = 10.03, *p* < .001 (see Fig 1). These results show that older adults were not averse to giving intentional explanations, but did so rather indiscriminately, with a lower level of intentionality attribution when appropriate (i.e., in the Cognitive videos), but a greater level when this was unsuitable, that is in the Random videos. This result is consistent with findings that older adults do worse on theory of mind tasks [11], and their difficulties were not simply linguistic because entering language abilities as a covariate did not change the pattern of results.

To summarize, the findings revealed a well-established age-related decline in ER and ToM, but older adults in the two conditions (high versus low self-involvement) performed similarly on both social-cognition tasks.

### Correlation analyses

Because participants in the Low and High self-involvement conditions did not differ on any variable, we collapsed across conditions and examined correlations within each age group as a whole. Our main aim was to examine whether individual differences in motivation correlated with performance. We separated young and older adults because they differed in both social-cognitive performance and motivation, and because motivation has been argued to play a more prominent role in later life.

First, we examined self-reported motivation, which was stable across the Matching and Animation tasks both for young and older adults (young adults: *r*(55) = .64, *p* < .001; older adults: *r*(59) = .48, *p* < .001), so that we collapsed it across the two task types. Self-reported motivation was not significantly related to social-cognitive performance, i.e., emotion recognition items and cognitive animation videos, in either age group, all *r*s ≤ .04, *p*s ≥ .764. Furthermore, overall no significant correlations emerged between self-reported motivation and performance on the non-emotion control items of the Matching task or on the control videos of the Animation task, *r*s ≤ .14, *p*s ≥ .285; the only exception to this pattern was a significant correlation between self-reported motivation and (inappropriate) intentionality in the Random videos for young adults only, *r*(55) = .29, *p* = .026. Overall, findings from the subjective measure of motivation indicated that older participants who reported higher levels of motivation did not perform better than those who claimed to be less motivated.

We then carried out an identical set of analyses for the physiological measure of motivation, SBP reactivity. As for the self-report measure of motivation, SBP-R was correlated across the Matching and Animation tasks for both for young and older adults, respectively, *r*(55) = .37, *p* = .004, and *r*(59) = .49, *p* < .001. Once again, then, we collapsed it across task type. SBP-R was not related to social-cognitive task performance in either age group, all *r*s ≤ .18, *p*s ≥ .173. Likewise, SBP-R was not associated with performance on the non-emotion control items of the Matching task or control videos of the Animation task, all *r*s ≤ .23, *p*s ≥ .078. Therefore, in neither age group did participants with higher engagement and effort mobilization (as indicated by SBP-R) perform better than participants with lower motivation.

We then computed Bayes factors for the primary correlations in an effort to examine whether current data offer sufficient evidence in favor of the null hypothesis of no relation between motivation and performance. Thus, we examined the association between (a) the two indices of motivation (self-reported and SBP-R) and (b) the two social-cognitive tasks (emotion recognition and cognitive videos). The results are summarized in Table 3. For two of the four correlations (the emotion recognition task) pertaining to older adults, we can accept the null hypothesis, H_0_, of no relation between motivation and performance. For the other two (the cognitive videos), the Bayes factors fell between .34 and 2.99, the region indicating that the data are insensitive for informing about either the H_0_ or H_1_. Similar results emerged for young adults.

**Table 3:** Pearson’s *r*, *p* values and bayes factors for correlation analyses among motivation and social-cognitive performance, separated by age group.

		Young			Older		
		*r*	*p*	*B*_U(0,0.549)_	*r*	*p*	*B*_U(0,0.549)_
**Self-reported motivation**	**Emotion recognition**	.02	.901	0.34	.01	.968	0.31
	**Cognitive videos**	-.01	.927	0.29	.04	.764	0.39
**SBP-R**	**Emotion recognition**	-.03	.812	0.26	.01	.918	0.33
	**Cognitive videos**	.04	.769	0.40	.18	.173	1.39

## Discussion

Three prior studies showed that increasing the familiarity of the stimuli or the experimenter improved the performance of older adults on tasks tapping social understanding [24–26]. Both sets of authors argued that familiarity may increase emotional meaningfulness and motivation (i.e., justified effort) in accomplishing such tasks, thus leading to better performance. However, in neither case did the authors actually measure motivation, nor did they examine non-social control tasks.

In the present study, we studied motivation in two ways. First, we attempted to manipulate motivation in the High self-involvement condition by telling participants that their performance on tasks tapping social-cognitive understanding was related to a range of desirable abilities and outcomes (e.g., positive social relationships), and that they would receive their results later; we then measured motivation using a self-report questionnaire and an objective physiological measure, namely systolic blood pressure change from baseline to within-task (SBP-R). Second, we examined correlations between motivation and task performance within age groups. If motivation is relevant to task performance, one would expect (a) older adults increasing their effort mobilization and engagement following the self-involvement manipulation, with positive effects on their performance, and (b) significant correlations between motivation and task performance. If, in contrast, motivation does not explain older adults’ impaired socio-cognitive performance, one would expect the self-involvement manipulation not to raise performance, nor the emergence of significant correlations. Our results were consistent with the latter possibility.

Although the self-involvement manipulation failed to increase motivation, we were still able to examine the role of motivation. On both the self-report and SBP-R measures of motivation, older adults were more (not less) motivated than young adults. This, therefore, is not consistent with their worse performance on the emotion recognition or theory of mind tasks. Then, when we examined within-group correlations, we found that motivation did not relate to task success in older adults either. Thus, we were able to examine the role of motivation directly (unlike previous researchers), yet we found no evidence supporting the idea that motivation is related to older adults’ worse social understanding. Indeed, older adults’ intact performance on the control items of the Matching task suggests that their worse performance on the emotion items was not related to motivation and effort mobilization, but reflects a specific decline in emotion recognition ability (i.e., older adults have selective difficulties, rather than general difficulties that could be explained by differences in motivation).

Before commenting in more detail on these core issues, there are some other findings that are worth mentioning. First, our results indicated a decline in emotion recognition and ToM, confirming existing research [1,11], and indicating that there was nothing unusual about our group of young and older adults, and hence, that the lack of a relation between motivation and task success was not due to the uniqueness of our participant group. Second, older adults were not averse to giving intentional explanations for the control items (Random videos) of the Animation task, but this was inappropriate, making their failure to give intentional explanations for the Cognitive items all the more interesting. This result suggests that older adults are aware that intentions guide behavior, but have difficulty assessing the circumstances for when intention ascription is most appropriate. Interestingly, a similar (though more severe) pattern of results has been found in atypical populations, such as people with autism and people with right hemisphere damage [68,69]. Relatedly, a recent study showed that older people’s lower ToM accuracy (compared to young adults), was due to both reduced ToM inferences as well as excessive errors in mental state attribution [70]. Third, the present results replicated earlier work, supporting the position that older adults generally mobilize more effort than young adults [22], as reflected in stronger SBP responses and greater self-reported motivation.

An unexpected result of the present study was the inefficacy of the self-involvement manipulation. Self-involvement refers to the personal importance attributed to a task, and the extent to which task performance is considered to reflect abilities considered as valuable by the subject [41]. We decided to manipulate self-involvement because in previous studies this motivation induction was associated with change in cardiovascular reactivity and because it positively impacted on the performance of young adults [36,60]. In previous studies young participants in the high self-involvement condition were told that higher performance was linked to a higher level of intelligence and better cognitive skills. However, we adapted the procedure in order to better fit with older adults’ goals, namely the preference for emotional well-being, personal meaningfulness, and positive and intimate social relationships [71,72]. Therefore, in the High self-involvement condition we linked better task performance with stronger social competence and functioning. Nevertheless, here the self-involvement manipulation was not associated with increased motivation whether measured by self-report questionnaire or by SBP-R during task performance. The question, then, is why our manipulation did not increase motivation. As stated above, previous studies have shown that young adult motivation has been increased by stating that performance is linked to higher intelligence and cognitive skills, but we adapted the task to be maximally motivating for older adults by focusing on emotional wellbeing and interpersonal relationships. This might explain the failure to increase performance in young adults, but not the failure to motivate older adults.

One possibility is that our manipulation unintentionally evoked age-related negative stereotypes in older adults, increasing stereotype threat and, thus, reducing cognitive performance. Notably, some research suggests that motivation and aging stereotypes interact in explaining older people’s cognitive performance [73]. Although we did not directly assess or manipulate aging stereotypes and thus can’t exclude this possibility, we argue that it is unlikely. First, stereotype threat affects performance in those abilities that are related to the stereotype, such as memory [74]. Second, if it was the case that our manipulation increased anxiety about performance (stereotype threat), then anxiety could work to facilitate performance (if it was moderate and helped to focus attention) or to impede performance (if it was severe). However, our data indicate that the motivation manipulation had no effect on task performance. Hence, there is no reason to think that the motivation manipulation elicited stereotype threat or that it impaired performance.

A different speculation is that participants in the experimental condition realized that the instructions were designed to increase motivation, yet did not believe them, impeding the effectiveness of the manipulation. Unfortunately, we did not check if participants were aware of the experimental manipulation, nor if they trusted what the instructions said, although we note that the information we provided was both accurate and plausible, and giving such instructions is a common practice in experimental aging research, with established reliability (e.g., [75,76]. Nevertheless, future studies could directly test this possibility, asking participants if they “guessed” the aim of the study and changed their behaviors/feelings accordingly.

We also note that our failure to find an effect due to a motivation manipulation is not without precedent. Koenig and Eagly [77] examined the effect of gender stereotypes on non-verbal communication decoding skill in young adults, and manipulated motivation by telling participants that the test predicts success in life and that they would receive feedback about their performance. The authors used a self-report questionnaire to measure motivation and found that neither self-reported motivation nor performance were affected by this manipulation. Given the difficulty of publishing null results, the fact that there is another such null result in the published literature lends credibility to our own similar finding.

Perhaps the most obvious reason of the failure to increase motivation is that older adults were already well motivated. In fact, this is the conclusion of several previous studies that have directly compared young and older adult motivation [39,40], and it is also supported by our own findings in the present study with both the self-report and the physiological measures of motivation indicating *higher* motivation in older adults. If correct, then older adults are already motivated to perform to the best of their ability, and their lower social understanding (compared to young adults) indicates a selective impairment that is not due to a lack of motivation, and thus cannot be improved by increasing effort mobilization.

Importantly, our claims about motivation are not limited to the self-involvement manipulation itself, so that the lack of an effect should not detract from the clear findings herein. That is, the present results are still highly interesting because they revealed that older adults were significantly *more* motivated than young adults (on both the SBP and self-report measures). Despite having *higher* motivation, older adults performed significantly worse on emotion recognition and ToM. This result is inconsistent with the idea that a lack of motivation causes older adults’ difficulties in social-cognition. Moreover, we note that older adults did not do worse on the control condition of the Matching task; if motivation was a general problem for task performance, one would expect that they would also do worse on this task, yet they did not. Furthermore, the correlational analyses showed that higher motivation was not associated with better social-cognitive performance in either young or older adults. For the emotion recognition tasks, the results were particularly clear in that the Bayes analysis indicated that current data offered substantial evidence in favor of the null hypothesis, i.e., that motivation is unrelated to emotion recognition understanding in older adults. In sum, even if the potential motivation manipulation was not successful, we could still provide clear analyses regarding the effect of motivation on social-cognitive performance.

Overall, the present findings are not consistent with the view that lower motivation negatively affects older adults’ social-cognitive performance [24–26]. It is important, once again, to acknowledge that previous researchers did not directly measure motivation, but instead, manipulated closeness or familiarity. However, we provided conjectural explanations for their findings that did not necessarily hinge on motivation, and, unlike previous researchers, we included two measures of motivation in our study: a self-report measure and a measure of systolic blood pressure reactivity. Despite measuring motivation in multiple ways, we found no link to performance and this was consistent over all tasks, both social and non-social.

### Cautions and conclusion

The present study has a number of limitations that should be acknowledged. First, the motivation manipulation did not affect motivation. We speculate that this is due to the fact that older adults were more motivated than young adults to begin with and it is difficult to raise motivation further. Nevertheless, other means of raising motivation could be attempted (although, again, we note that older adult motivation did not correlate with task success). We believe that present findings should encourage future studies to explore different means of operationalizing and manipulating motivation.

We also note that nearly 50% of older participants reported taking anti-hypertensive medications. Although this percentage might seem high and might seem to necessitate a caution, it is in fact entirely representative of the general population [78]. Thus, once again, there was nothing unusual about our participants. In addition, we note that blood pressure reactivity (i.e., the increase in SBP) was no different in the group on hypertensive medication relative to the group not on medication, the results were similar when we excluded participants with a decrease in SBP-R from baseline, and that the self-report questionnaire yielded similar findings to SBP-R.

Third, we did not explicitly assess or manipulate the perceived difficulty of the tasks. As stated in the Introduction, the motivational intensity theory predicts that effort is mobilized as a result of both motivation and the perceived difficulty of the task [42]. In the present study, we assumed that both social-cognitive tasks were not too easy, with support for this assumption coming from the fact that only 1.8% of participants got the maximum score in the ToM and ER tasks. Moreover, in neither task did we provide feedback, thereby leaving participants uncertain as to their success. However, future studies should directly assess perceived task difficulty, given its moderating role in the interplay between self-involvement and effort mobilization.

Fourth, the present results need to be replicated in a larger sample and, we believe, cross-cultural studies are especially needed to examine if different types of motivation manipulation differently affect older people’s social-cognitive performance depending on their cultural values and goals.

In sum, the present study contributed to research examining the role of motivation and social understanding in aging. For the first time, we provide direct indices of motivation, by using both self-report and cardiovascular reactivity measures of motivation. This was done to overcome a common limitation of subjective measures, namely, that self-reported motivation may not reflect actual effort and engagement [79]. The findings converge in showing that older adults were more motivated than young adults on both measures of motivation. Also, the findings revealed that, despite their higher effort mobilization, older adults’ emotion recognition and theory of mind was worse, yet they were not worse compared to young adults on the non-emotion control items of the Matching task. Finally, the current study is the first showing that individual differences in motivation did not correlate with ER or ToM performance.

Overall, our results were not consistent with previous claims that lower motivation results in older adults’ worse social cognition [24–26]. In contrast, our results were consistent with the idea that older adults experience a decline in social understanding [1,11], and with the claim that they are highly motivated on such tasks [39,40]. Our findings suggest that the relation between motivation and performance is far from straightforward; high motivation is not sufficient for better performance.

## Supporting information

S1 Appendix Instructions given to participants in the High vs. Low self-involvement conditions. (DOCX)

S1 Dataset Final sample (*n* = 118) used for main analyses. (XLSX)

S1 File Analyses on diastolic blood pressure (DBP) and heart rate (HR). (DOCX)
