# Supplementary material for: Motivation and social-cognitive abilities in older adults: Convergent evidence from self-report measures and cardiovascular reactivity
Source: PLoS One. 2019 Jul 10;14(7):e0218785. doi: 10.1371/journal.pone.0218785 (PMC6619662; doi:10.1371/journal.pone.0218785)
Supplement: S1 Appendix — (DOCX) [file pone.0218785.s001.docx]

**S1 Appendix. Instructions given to participants in the High vs. Low self-involvement conditions.**

| **Matching task** | |
| --- | --- |
| *High self-involvement* *condition* | *Low self-involvement condition* |
| “[…] Previous research has shown that people who have a high score in this task usually are also highly socially competent. For example, they are able to discriminate a lie from a truth, they are better and more enjoyable communicators, and they are better at recognizing when someone has made a social gaffe. At the end of the experiment, I will give you your results so you can check your skill.” | “[…] Previous research using this task are been carried out in a very wide range of countries. The voices and the sounds were recorded in this laboratory from volunteers who helped us or were retrieved on the internet. The faces were drawn from an international set of faces and they are commonly used in psychology research. At the end of the experiment, I will give you another kind of task.” |
| **Animation task** | |
| *High self-involvement condition* | *Low self-involvement condition* |
| “[…] According to several researchers, high scores in this task are related to better relations between people and to the ability to make and keep good friendships. High scores are also associated with social competence and altruistic attitudes and are considered markers of wisdom. At the end of the experiment, I will give you your results, so you can check your skills.” | “[…] This task has been first used in 1944 by Heider and Simmel in the USA. Years later, other researchers rediscovered and adapted it and now it is used in research worldwide. This task has been adapted from a previous version with different geometrical shapes, like circles and squares. At the end of the experiment, I will give you another kind of task.” |
