# Supplementary material for: Motivation and social-cognitive abilities in older adults: Convergent evidence from self-report measures and cardiovascular reactivity
Source: PLoS One. 2019 Jul 10;14(7):e0218785. doi: 10.1371/journal.pone.0218785 (PMC6619662; doi:10.1371/journal.pone.0218785)
Supplement: S1 File — (DOCX) [file pone.0218785.s003.docx]

**S1 File.** **Analyses on diastolic blood pressure (DBP) and heart rate (HR).**

Preliminary analyses confirmed that blood pressure medication consumption did not impact on DBP and HR reactivity, *F*(3, 57) = 1.02, *p*= .392 and *F*(3, 57) = 1.98, *p*= .127, respectively.

Descriptives are reported in Table S1. A series of 2 (Age Group: young, older) x 2 (Condition: high self-involvement, low self-involvement) ANOVAs were performed to investigate potential differences in DBP and HR values both at baseline and during task performance (i.e., reactivity). Results are presented in TableS1.

Table 1. Means (and standard deviations) separated for age group and condition. The last three columns present results from ANOVAs, reporting Fisher’s *F* and *p* for both main and interaction effects.

|  | **Young** | | **Older** | | **ANOVA** | | |
| --- | --- | --- | --- | --- | --- | --- | --- |
|  | *Low self-involvement* | *High self-involvement* | *Low self-involvement* | *High self-involvement* | *Age* | *Condition* | *Age x Condition* |
| DBP-base | 73.90  (6.02) | 70.63  (8.44) | 78.60  (9.45) | 77.64  (10.20) | ***F =* 13.20**  ***p* < .001** | *F =* 1.73  *p* = .191 | *F =* 0.51  *p* = .476 |
| HR- base | 75.27  (9.62) | 77.94  (11.65) | 68.05  (7.57) | 75.96  (12.16) | ***F =* 5.69**  ***p* = .019** | ***F =* 7.51**  ***p* = .007** | *F =* 1.84  *p* = .177 |
| *Matching task* | | | | | | | |
| DBP-R | 1.75  (6.45) | 1.28  (5.36) | 0.69  (7.83) | 2.54  (5.55) | *F =* 0.01  *p* = .930 | *F =* 0.35  *p* = .558 | *F =* 0.99  *p* = .323 |
| HR-R | 1.55  (8.20) | 1.69  (7.63) | - 1.20  (5.54) | - 0.86  (6.57) | ***F =* 4.17**  ***p* = .043** | *F =* 0.33  *p* = .856 | *F =* 0.01  *p* = .942 |
| *Animation task* | | | | | | | |
| DBP-R | 3.64  (11.45) | 3.16  (7.45) | 6.06  (9.89) | 3.52  (8.50) | *F =* 0.65  *p* = .423 | *F =* 0.76  *p* = .386 | *F =* 0.36  *p* = .552 |
| HR-R | 3.01  (7.46) | 1.24  (9.08) | - 0.72  (5.32) | - 3.29  (6.75) | ***F =* 9.51**  ***p* = .003** | *F =* 2.62  *p* = .108 | *F =* 0.09  *p* = .766 |

*Note.* DBP-base = diastolic blood pressure during the baseline. HR-base = heart rate during the baseline. DBP-R = diastolic blood pressure reactivity. HR-R = heart rate reactivity.

Correlation analyses examined the relationships between DBP and HR reactivity (collapsed across the two tasks) and social-cognitive performance. Results are presented in Table S2.

Table 2. Pearson’s *r* (and *p*) investigating the correlation between DBP and HR reactivity and socio-cognitive performances separated for young and older participants.

|  | **Young** | | **Older** | |
| --- | --- | --- | --- | --- |
|  | DBP-R | HR-R | DBP-R | HR-R |
| *Matching task* | | | | |
| Emotion recognition | .05  (.727) | .02  (.878) | -.01  (.967) | -.26  (.051) |
| Non-emotion control | .02  (.869) | -.03  (.833) | .19  (.149) | .08  (.537) |
| *Animation task* | | | | |
| Cognitive | -.01  (.943) | -.11  (.441) | .18  (.160) | -.14  (.297) |
| Goal-directed | **-.32**  **(.016)** | -.23  (.086) | .13  (.349) | -.08  (548) |
| Random | -.10  (.460) | -.05 (.719) | .05  (.719) | -.02  (.856) |

*Note.* DBP-R = diastolic blood pressure reactivity. HR-R = heart rate reactivity.
